# Longitudinal study of electrical, functional and structural remodelling in an equine model of atrial fibrillation

**DOI:** 10.1186/s12872-019-1210-4

**Published:** 2019-10-21

**Authors:** Eva Zander Hesselkilde, Helena Carstensen, Mette Flethøj, Merle Fenner, Ditte Dybvald Kruse, Stefan M. Sattler, Jacob Tfelt-Hansen, Steen Pehrson, Thomas Hartig Braunstein, Jonas Carlson, Pyotr G. Platonov, Thomas Jespersen, Rikke Buhl

**Affiliations:** 1 Department of Veterinary Clinical Sciences, Faculty of Health and Medical Sciences, University of Copenhagen, Højbakkegaard Allé 5, 2630 Taastrup, Denmark; 2 Department of Biomedical Sciences, Faculty of Health and Medical Sciences, University of Copenhagen, Blegdamsvej 3, 2200 Copenhagen, Denmark; 3 Department of Cardiology, The Heart Centre, Copenhagen University Hospital, Blegdamsvej 9, 2100 Copenhagen, Denmark; 4 Department of Medicine I, University Hospital Munich, Campus Grosshadern, Ludwig-Maximilians University Munich (LMU), Munich, Germany; 5 Department of Forensic Medicine, Faculty of Health and Medical Sciences, University of Copenhagen, Frederik V’s vej 11, 2100 Copenhagen, Denmark; 6 Department of Cardiology, Clinical Sciences, Arrhythmia Clinic, Skåne University Hospital, Lund University, 21185 Lund, Sweden

**Keywords:** Animal model, Atrial fibrillation, Atrial fibrillatory rate, Chronic atrial fibrillation, Equine, Flecainide, Horse

## Abstract

**Background:**

Large animal models are important in atrial fibrillation (AF) research, as they can be used to study the pathophysiology of AF and new therapeutic approaches. Unlike other animal models, horses spontaneously develop AF and could therefore serve as a bona fide model in AF research. We therefore aimed to study the electrical, functional and structural remodelling caused by chronic AF in a horse model.

**Method:**

Nine female horses were included in the study, with six horses tachypaced into self-sustained AF and three that served as a time-matched sham-operated control group. Acceleration in atrial fibrillatory rate (AFR), changes in electrocardiographic and echocardiographic variables and response to medical treatment (flecainide 2 mg/kg) were recorded over a period of 2 months. At the end of the study, changes in ion channel expression and fibrosis were measured and compared between the two groups.

**Results:**

AFR increased from 299 ± 33 fibrillations per minute (fpm) to 376 ± 12 fpm (*p* < 0.05) and atrial function (active left atrial fractional area change) decreased significantly during the study (p < 0.05). No changes were observed in heart rate or ventricular function. The AF group had more atrial fibrosis compared to the control group (p < 0.05). No differences in ion channel expression were observed.

**Conclusion:**

Horses with induced AF show signs of atrial remodelling that are similar to humans and other animal models.

## Background

Atrial fibrillation (AF) remains the most important arrhythmia in clinical practice, with a high morbidity, mortality and economic burden on society (1). AF alters the electrophysiology, the structure and the contractility of the atrium – a process commonly known as atrial remodelling. Atrial remodelling further promotes AF (2) and impedes cardioversion to sinus rhythm (SR). Animal models complement clinical studies by investigating the underlying mechanisms leading to AF and its maintenance. Furthermore, a good large animal model could also indicate whether new antiarrhythmic drugs will be useful in the clinic and report on potential side effects. Models for AF research span ex vivo cellular studies to larger animal models, yet the preference seems to have shifted over the years toward: 1) large animal models, as the AF mechanisms are highly complex and require a proper substrate and 2) chronic AF models, as AF develops over time and these changes are crucial for optimal treatment (3).

Horses fulfil several requirements of a *bona fide* model for AF studies; horses are large animals that are easy to handle and many procedures can be performed without sedation or anaesthesia. They have a high AF inducibility (4, 5), and most importantly, spontaneously develop AF both with and without the presence of structural heart disease. Furthermore, horses with chronic AF do not develop heart failure, which facilitates long-term studies (6). Horses have previously been suggested as an animal model for AF studies and several studies have investigated the short term effect of induced AF in horses (15 min – 7 days) (4, 5, 7–9) while only a few studies on long-term AF exist (10, 11).

In order to establish the horse as an animal model, it is important to document the effect of AF on electrical, structural and contractile remodelling over time. The aim of the study was therefore to develop a long-term (55 days) horse model with tachypacing-induced AF. Electrocardiography (ECG), echocardiography, histology and molecular expression studies were performed in order to evaluate temporal changes. We hypothesized that horses with induced AF would develop electrical, functional and structural remodelling, observed as changes in atrial fibrillatory rate (AFR), reduced atrial function and changes in ion channel expression and atrial fibrosis, respectively.

## Methods

Nine female Standardbred horses aged 4–17 years (10 ± 5 years), with a body weight between 408 and 572 kg (474 ± 47 kg) were included in the study. The horses were purchased as experimental animals from private trainers. Six horses were electrically stimulated into AF (the AF group) and three served as a time-matched, sham-operated control group. All animals were deemed healthy based on clinical examination including blood analysis, and no abnormalities were found from ECG or echocardiographic examinations at the time of inclusion.

### Pacemaker implantation

Pacemaker implantation in horses has previously been described (12). In this study dual-chamber pacemakers^1^ were implanted in nine horses for AF induction (AF group) and electrophysiological studies (AF and control group). The pacemakers were implanted in sedated (0.01 mg/kg detomidine, 0.01 mg/kg butorphanol and constant rate infusion of 1.0 mg/ml xylazine) standing horses. The pectoral region was surgically prepared and locally anesthetised before a 6–8 cm incision was made. Through the cephalic vein two leads^2^ were advanced to the heart and both were fixated in the right atrium. Pacing ability, threshold, lead impedance and absence of phrenic nerve stimulation were tested before and after fixation. The leads were secured in the cephalic vein and connected to the pacemaker which was placed in a subcutaneous pocket distal to the incision. The incision was sutured in three layers and the horses were treated with antibiotics and non-steroidal anti-inflammatory drugs. The surgery-time was between 60 and 150 min.

### AF induction and study design

**Fig. 1:**
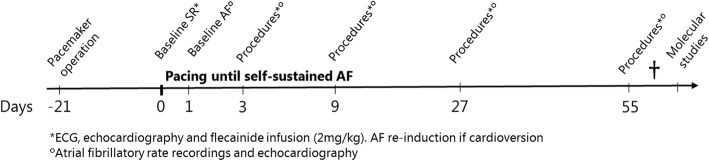
Study design. Study start at day 0, three weeks after pacemaker implantation. Baseline measurements were performed in sinus rhythm (baseline SR) before atrial fibrillation (AF) induction and 24 h after pacing was initiated (day 1, baseline AF). Pacing continued until self-sustained AF. Experimental protocol included electrocardiography (ECG), echocardiography, atrial fibrillatory rate recordings (AF group) and flecainide administration. Cardiac tissue was harvested at the end of the study

The study design is depicted in Fig. 1. AF was induced by high-rate pacing from the pacemaker, pacing from both leads at a rate of 170 min^− 1^ (with a delay between the leads of 175 ms and 2.5 V @ 0.4 ms output) which resulted in an atrial pacing rate of 340 min^− 1^. AF was initiated by burst-pacing operated manually through the pacemaker, which was continued daily (1–8 h per horse) until sustained AF was achieved. During AF (in the period of AF induction), the pacemaker was in an inhibitory mode to automatically start pacing if the atrial rate fell below 170 min^− 1^, thus ensuring a minimum atrial rate of 170 min^− 1^ at all times. The horses were constantly monitored with ECG during AF induction, except for a few hours per day, when they were allowed to walk freely in the paddock. The pacemaker was turned off after the horses had been in self-sustained AF for more than 24 h.

Total AF durations were calculated from the surface ECG, and all AF episodes of more than 10 min were included. Episodes shorter than 10 min, burst-pacing and pacemaker activity were not included. If the horses spontaneously cardioverted to SR in the paddock (when not equipped with an ECG) or at night (after dislodging the electrodes), these periods were registered as uncertain AF and were not included in the total AF duration.

To investigate the effect of both short- and long-term AF on atrial remodelling, the procedures described below were performed 3, 9, 27 and 55 days (procedure days) after pacing was initiated. Baseline measurements were obtained on day 0 before pacing was initiated (SR measurements) and on day 1 (AF measurements). If the horses were not in AF on the procedure days, the pacemaker was turned off and the procedures were performed in SR. To study the effect of remodelling on pharmacological cardioversion, intravenous flecainide^3^ (2 mg/kg, rate 0.2 mg/kg/min) was administered on each of the procedure days. Flecainide was chosen for cardioversion as it previously has been studied in horses, it is used in the clinic to treat paroxysmal AF and has a short half-life (7). The pacemaker was turned off at least 30 min before flecainide infusion began to avoid that spontaneous cardioversion was attributed to the effect of flecainide. A detailed description of the effects of flecainide can be found elsewhere (13). At the end of each procedure day, AF was re-induced following the same induction protocol as described above.

### Electrocardiographic parameters

During AF induction and throughout each procedure day, the horses were equipped with a three-lead Holter ECG^4^ with the electrodes placed as a modified base-apex ECG (7). The ECGs from each procedure day were manually analysed offline by a single observer; QRS duration and QT interval were measured and averaged over five consecutive beats and HR was averaged over 10 beats. QT measurements were adjusted beat-to-beat according to species specific HR (QTc), as previously described (14).

### Atrial fibrillatory rate

Atrial fibrillatory rate (AFR) is a non-invasive measurement of atrial remodelling, and is inversely proportional to atrial fibrillation cycle length (AFCL) (15). AFR was calculated from a 15-min surface ECG recording of AF. ECG analysis including QRST cancellation and AFR calculation was performed using the Cardiolund AFR Tracker software^5^ (16).

### Functional studies

Echocardiographic examinations were performed to quantify left atrial and ventricular size and function. All echocardiographic examinations were performed using a portable Vivid I ultrasound system with a 3.1 MHz phased array transducer^6^ at rest and without sedation, as previously described (17). Echocardiographic examinations were performed before pacemaker implantation, at baseline and on all procedures days (before, days 0, 1, 3, 9, 27 and 55) in both SR and AF when possible. Horses in the AF group were examined in AF before administration of flecainide, and an additional examination was performed if they cardioverted to SR after flecainide treatment. Assessment of ventricular size and function was performed from the examinations obtained in AF while assessment of atrial size and function was performed from the SR examinations as the left atrial fractional area change measurements only can be obtained in SR. Horses in the control group were examined before flecainide administration. Colour flow Doppler was used to assess valvular competences. Analysis was performed offline^7^ by a single observer and two-dimensional echocardiography (2D) and anatomical M-mode (AMM) were used to assess the left atrial (LA) and left ventricular (LV) size and function. Values are reported as the average of three non-consecutive cardiac cycles. Ventricular measurements are described in the Additional file 1. Two-dimensional echocardiographic variables for the assessment of LA size and function were measured as follows: left atrial area at mitral valve closure (LAA_min_), left atrial area at onset of the P wave (LAA_a_), left atrial area at mitral valve opening (LAA_max_), left atrial diameter at mitral valve opening (LAD), passive left atrial fractional area change (LA-Fac_passive_ = (LAA_max_ – LAA_a_) / LAA_max_), active left atrial fractional area change (LA-Fac_active_ = (LAA_a_ – LAA_min_) / LAA_a_) and total left atrial fractional area change (LA-Fac_total_ = LAA_max_ – LAA_min_) / LAA_max_) (18). Valvular insufficiency was classified as: none, clinically insignificant, mild, moderate or severe (19).

### Structural studies

The horses were euthanized between 58 and 63 days after AF initiation by bolt-stunning, and cardiac tissue was harvested for microscopy and quantitative polymerase chain reaction (qPCR, described in Additional file 1: Table S1) from the left atrial appendage (LAA), right atrial appendage (RAA) and right ventricle (RV). Biopsies for microscopy were sliced (4 μm) and stained with Picro-Sirius Red. Detailed information about the preparation of the tissue can be found in the Additional file 1. Full biopsy sections were scanned, using a ZEISS Axioscan slide scanner equipped with a 20x, 0.8 NA objective.^8^ Scanned images were analysed with the software ZEN 2.3 Blue edition^9^ in combination with the extension software ZEN Intellesis,^10^ which was used for image segmentation to distinguish between background, muscle and collagen. We applied two approaches for collagen assessment. First, the full sections including large vessels, epi- and endocardial tissue were analysed. Second, quantification of interstitial fibrosis, where three areas from each section containing either high amounts (interstitial_max_) or low amounts (interstitial_min_) of collagen between the cardiomyocytes were chosen, by eye and blindfolded to the observer, and analysed as the average of interstitial_min_ and interstitial_max_.

### Data analysis

Investigators were blinded to the group allocation and procedure day throughout all analyses. Data analyses were performed using Microsoft Excel^11^ and GraphPad Prism.^12^ Changes in AFR and other ECG parameters between baseline and day 55, were assessed using student’s t-tests where paired analysis were performed when appropriate. Difference in fibrotic area and ion channels expression (measured with qPCR) between AF and control were also assessed using student’s t-test. The qPCR data were subsequently Bonferroni corrected. Echocardiographic data were compared to baseline measurement with a one-way ANOVA followed by Dunnett’s post hoc test for multiple comparisons. Data are presented as mean ± SD and *p* ≤ 0.05 was considered significant.

## Results

All horses completed the study period of 55 days. One control horse developed non-cardiac-related colic and could not participate on procedure day 3. After the study period (day 59), cardioversion was attempted in horse #1 with an additional dose of flecainide 10 min after the first dose. The horse developed ventricular fibrillation and was not included in the molecular studies.

### AF induction

**Fig. 2:**
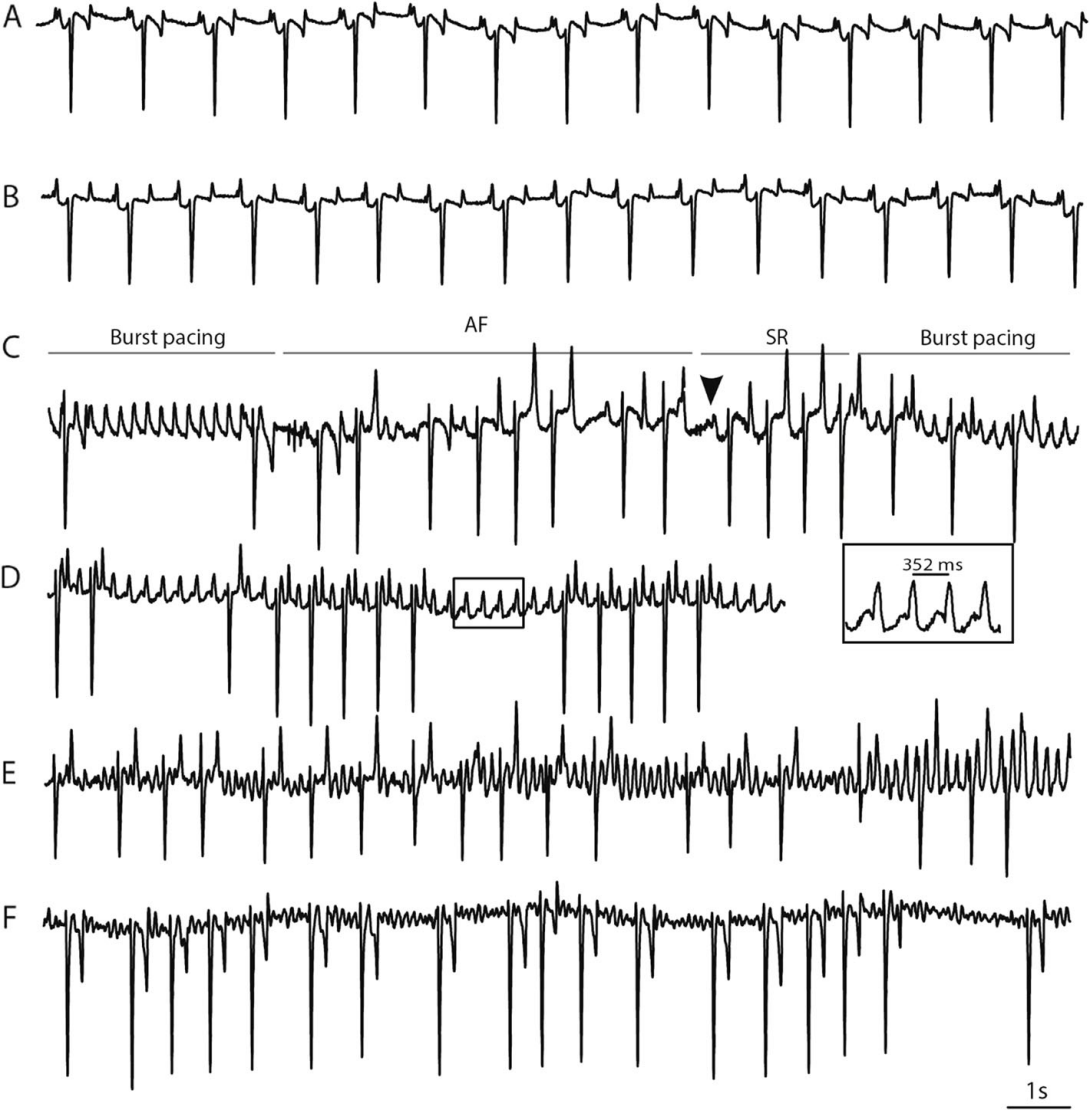
ECG traces during the study. A: Control horse in SR at day 0 and at day 55 (B). C: Manual burst-pace at day 0 resulted in short AF episodes followed by SR. Arrow head indicate the first P wave after spontaneous cardioversion. D: Pacemaker driven atrial rhythm of 170 min^− 1^ (352 ms) with intermittent AV block. E and F: One horse in AF at day 1 and day 55, respectively. Note the faster fibrillatory rate at day 55 (F). Paper speed 10 mm/s, gain 10 mm/mv

**Fig. 3:**
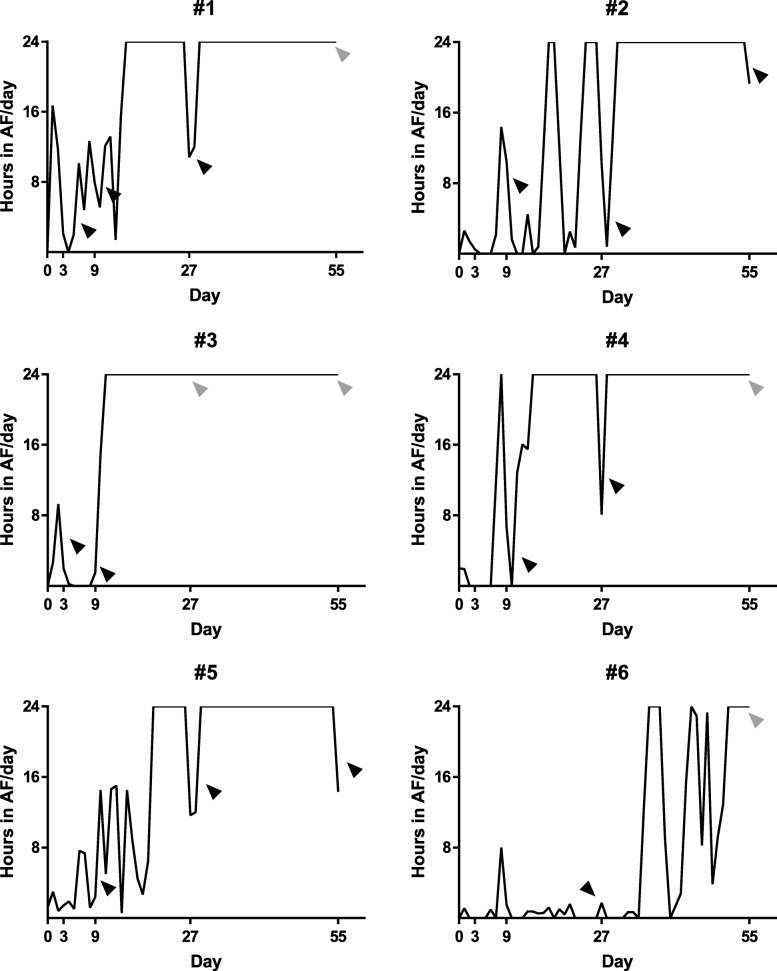
AF burden. Total AF burden for individual horses. Black arrowheads indicate successful cardioversion to sinus rhythm after flecainide administration. Grey arrowheads indicate unsuccessful cardioversion after flecainide administration. Pacing, AF episodes < 10 min and unknown AF are not included

All horses were continuously paced into self-sustained AF until they no longer spontaneously cardioverted to SR. The pacing time required to achieve self-sustained AF varied among horses (18 ± 9.9 days, range 8–36 days). AF stability increased over time for all horses; at the beginning of AF induction, burst-pacing only resulted in short AF episodes, but as pacing continued, longer AF episodes occurred and eventually only a small number of burst-pacing sessions were needed for AF to become sustained (Fig. 2, panel C). The AF became less organized over time as it developed into more stable AF (Fig. 2, panel E and F). In contrast, no ECG changes were observed in the control group between day 0 and day 55 (Fig. 2, panel A + B). Total AF duration ranged from 15 to 46 days (37 ± 11 days), and the AF burden for each horse is illustrated in Fig. 3. The total duration of unknown AF was on average 2.4 days per horse and was not included.

### Electrical remodelling

#### ECG changes

There was a trend towards an increased HR in the AF group from day 0 (measured in SR) to day 55 when the horses were in AF (HR day 0: 42 ± 13 min^− 1^, HR day 55: 64 ± 20 min^− 1^, *p* = 0.07). No difference in HR was observed for the control group (HR day 0: 46 ± 10.2 min^− 1^, day 55: 42 ± 7 min^− 1^, *p* < 0.05). No changes in QRS or QTc duration were observed in any of the groups (p < 0.05). In the AF group, QRS duration was 103 ± 5 ms on day 0 and 100 ± 7 ms on day 55. In the control group, QRS duration was 93 ± 3 on day 0 and 92 ± 6 on day 55. Mean QTc duration was 442 ± 38 ms on day 0 and 441 ± 23 ms on day 55 for the AF group and 438 ± 1 ms on day 0 and 458 ± 10 ms on day 55 for the control group.

#### Atrial fibrillatory rate

**Fig. 4:**
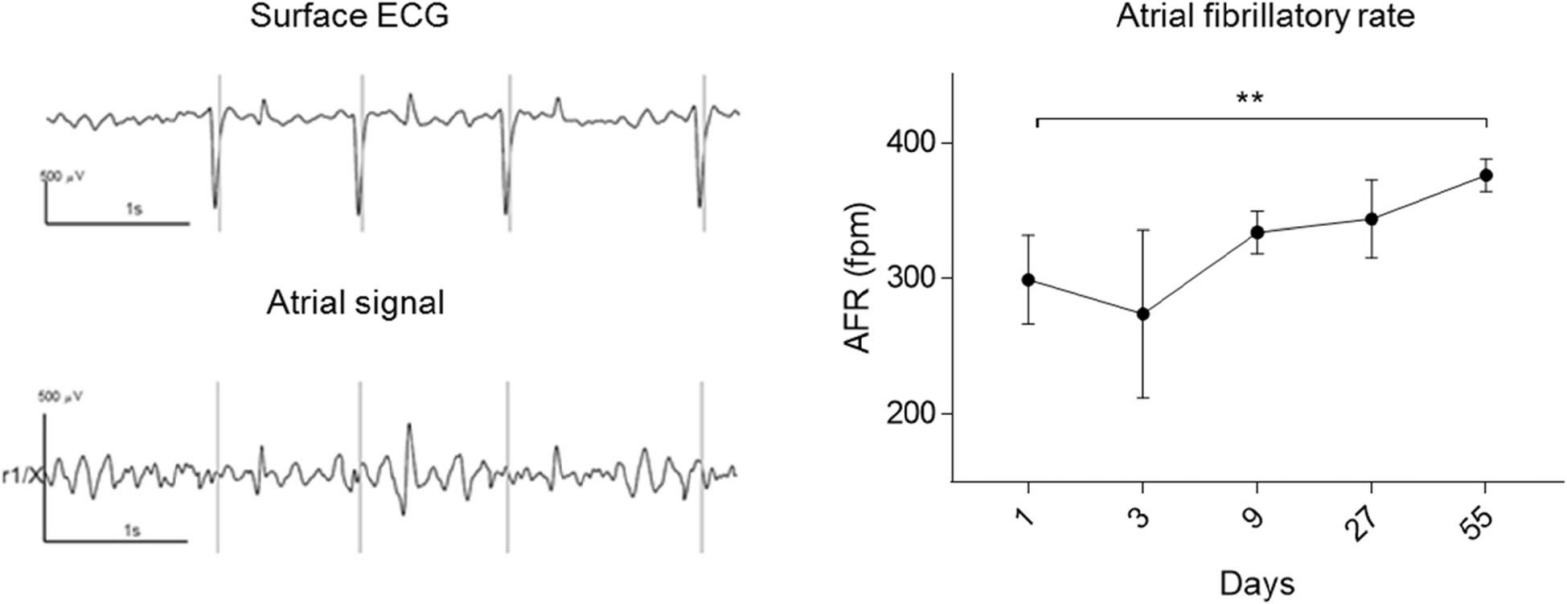
Atrial fibrillatory rate. Left: Example of spatiotemporal QRST cancellation: From the surface ECG (top trace), the software identifies the QRS complexes. After QRST cancellation, only the atrial signal remains (bottom trace). Right: The course of AFR during the study. AFR increases significantly (*p* < 0.01) from day 1 to day 55 consistent with atrial remodeling. *n* = 6 except day 3 (*n* = 2). Data are presented as mean ± SD. AFR = atrial fibrillatory rate, fpm = fibrillations per minutes. Asterisks indicate statistical significance (p < 0.01)

On day 1 (24 h after pacing was initiated), AFR was 299 ± 33 fibrillations per minute (fpm; range 239–323 fpm). The AFR increased during the study (apart from day 3 when only two horses were in stable AF) and was significantly higher on day 55 compared to day 1 (mean AFR on day 55: 376 ± 12 fpm, range 362–394 fpm, *p* < 0.01, Fig. 4).

#### Ion channel expression

No significant changes in ion channel expression were found for any of the genes (SCNA5, CACNA1C, KCND3, KCNIP2, KCNA5, KCNH2, KCNQ1, KCNJ2, KCNJ3, KCNJ5 and KCNN1–3.) between the AF and control horses. Expression levels for each gene are presented in Additional file 1: Fig. S1.

### Functional remodelling

Offline assessment of echocardiographic image and data quality led to the exclusion of ventricular measurement obtained in AMM for two horses from the AF group (on day 1 and 55), and ventricular measurement obtained in 2DE for 1 horses in the AF group at day 55 and for atrial images in one horse during AF at day 1.

**Fig. 5:**
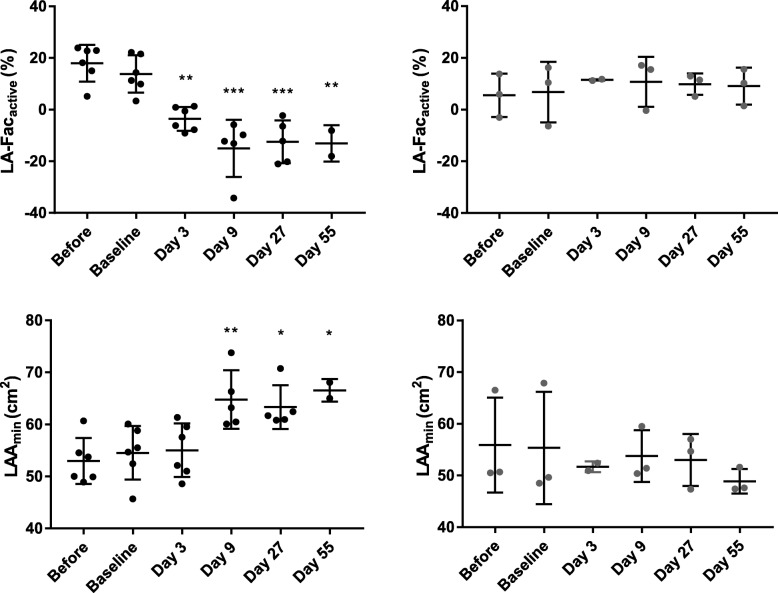
Functional remodelling of the left atrium. Echocardiographic evaluation of atrial size and function. Top panel illustrates the left atrial active fractional area changes (LA-Fac_active_) in the AF group (left) and in the control group (right) before pacemaker implantation (before), at baseline (day 0) and on each procedure day (day 3, 9, 27, and 55). Bottom panel illustrates the left atrial area at mitral valve closure (LAA_min_). Asterisk indicate significant differences from baseline (*, *p* < 0.05, **, p < 0.01, ***, *p* < 0.001). All echocardiographic examinations were obtained in SR after cardioversion with flecainide (AF group). Data are presented as mean ± SD

From the echocardiographic examinations performed in SR after cardioversion with flecainide (AF group) we found that the left atrial area (LAA_min_) significantly increased after day 9 (*p* < 0.01, Fig. 5) compared to baseline while no significant changes were observed for LAA_max_ or LAD. The LA-Fac_active_ significantly decreased after day 3 (p < 0.01, Fig. 5) while no significant changes were observed for LA-Fac_passive_ but a tendency for LA-Fac_total_ was found at several time points and showed significant difference at day 27 (p < 0.01). No differences were observed between examinations performed before pacemaker implantation and at baseline (after pacemaker implantation, before induction of AF). Before cardioversion with flecainide the horses in the AF group had an echocardiographic examination while in AF. Data from these examinations revealed a significant enlargement of the LAA_min_ and LAA_max_ between baseline (AF baseline = day 1) and day 55 (p < 0.01). No changes in LAD or LA-Fac_total_ were observed. Measurement of LAA_a_, LA-Fac_active_ and LA-Fac_passive_ were not possible as no P wave was visible during AF. We observed no changes in either atrial size or function in the control group (Fig. 5 and Additional file 1: Table S2). Measurements of atrial size and function before the pacemaker was implanted, at baseline, during the AF episodes and after cardioversion are illustrated in Additional file 1: Table S2.

Colour flow Doppler revealed a moderate tricuspid valve insufficiency in one horse at inclusion that did not progress during the study. Several horses had mild and clinically insignificant valve insufficiencies. None of the horses developed valvular insufficiencies beyond the “mild” category throughout the study.

To study ventricular remodelling and monitor signs of heart failure, LV size and function were in each group assessed during the study. No differences were observed in the AF or control groups during the study period (*p* > 0.05). Results are displayed in Additional file 1: Table S3–4.

### Cardioversion to sinus rhythm

Results on cardioversion rates with flecainide have previously been published (13). Briefly, one horse was in stable AF on procedure day 3, and cardioverted 3 min after flecainide infusion was initiated. On day 9, 5/5 horses cardioverted (6–12 min after flecainide infusion began), on day 27, 5/6 horses cardioverted (4–62 min after infusion began) and on day 55, 2/6 horses cardioverted (96 and 185 min after infusion began, Fig. 3). There was a correlation between the time from infusion start until cardioversion to SR and the cumulative duration of AF (*p* < 0.001, R^2^ = 0.80) (13).

### Structural remodelling

**Fig. 6:**
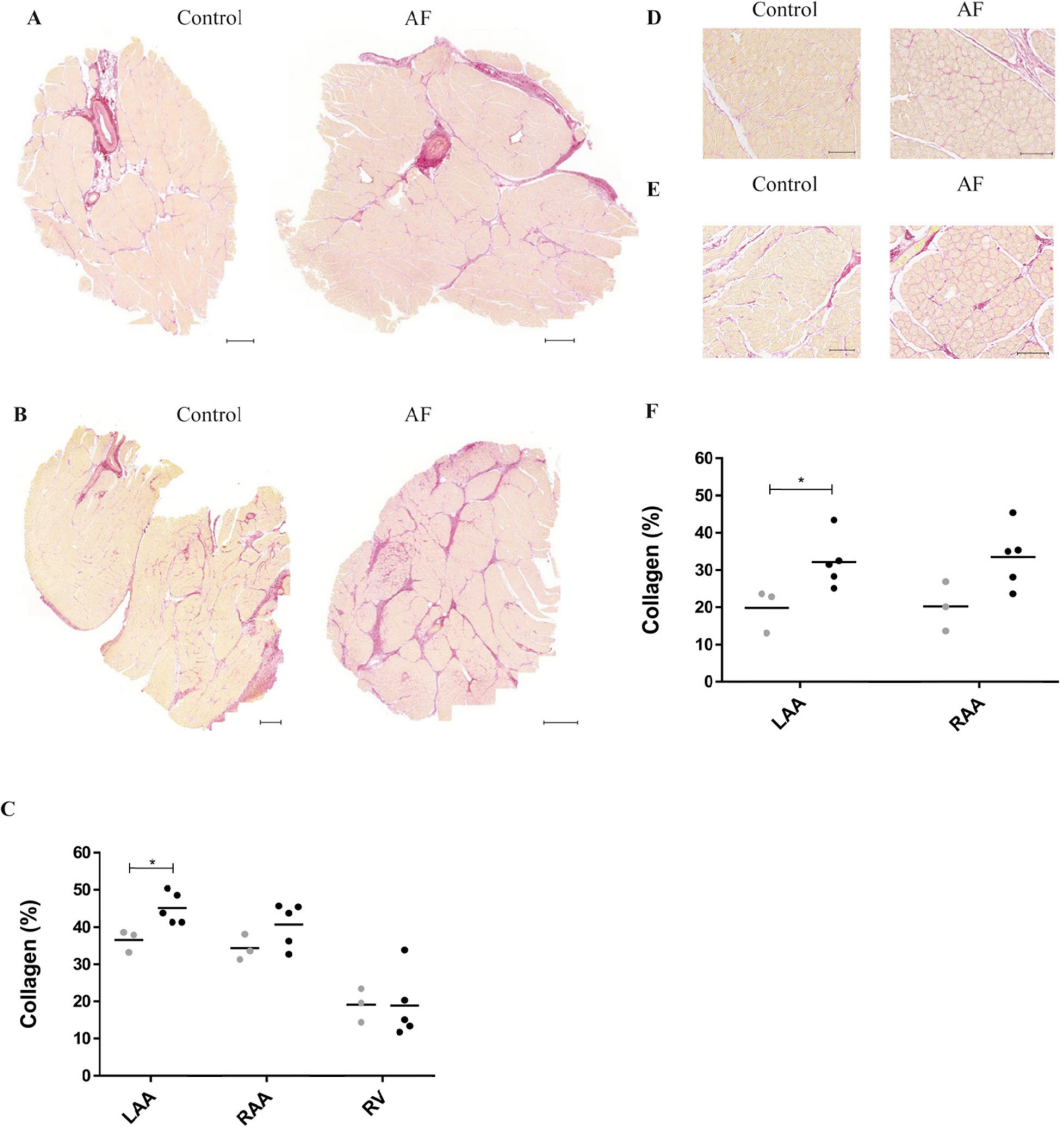
Fibrosis. Picro-Sirius Red staining of cardiac tissue from the AF group (*n* = 5) and the control group (*n* = 3). A + B: Representative images of left atrial appendage (LAA, panel A) and right atrial appendage (RAA, panel B) sections, respectively. Fibrosis is stained red. C: Quantification of Picro-Sirius Red positive areas for full sections of the three regions: LAA, RAA and right ventricle (RV). Collagen was significantly increased in the LAA in the AF group (black) compared to the control group (grey). D + E: Representative images showing areas of interstitial fibrosis for LAA (D) and RAA (E). F: Quantification of Picro-Sirius Red positive areas showed a significant increase in interstitial fibrosis for the AF group in the LAA compared to the control group. Sections for RV not shown. *p < 0.05. Scale bar = 500 μm in (A and B) and 100 μm in (D and E)

With the Picro-Sirius Red staining of connective tissue we found a significantly increased collagen deposition in the left atria in the AF group (compared to the control group) for both the full LAA sections (*p* < 0.05, Fig. 6A, C) and the analysis of interstitial fibrosis (p < 0.05, Fig. 6D, F). There were no significant differences found for the full RAA section (*p* = 0.14, Fig. 6B, C), although a tendency was seen in the analyses of interstitial fibrosis (*p* = 0.06, Fig. 6E, F). Quantification of collagen deposition of the RV sections (Fig. 6C) showed no differences between the two groups (*p* = 0.97).

## Discussion

In this study, we have introduced a novel model of chronic AF. We found that horses can be tachypaced into to self-sustained AF and that AF promotes electrical, functional and structural remodelling. Horses spontaneously develop AF both with and without the presence of underlying cardiac disease, which is why we present the horse as a highly relevant animal model of human AF. In order to study changes from early AF to chronic AF, we included healthy horses, in which AF was induced by tachypacing.

### AF induction

The pacemaker ensured a constant rate between 170 and 340 min-1 in the atria, however this did not lead to sufficient capture nor the development of self-sustained AF. Burst-pacing was therefore introduced and combined with pacing from the pacemaker it proved feasible for inducing self-sustained AF in horses. Other animal studies (8, 20) have used neurostimulators to automatically burst-pace the atria which is desirable as it is less time-consuming for the operators and ensures a uniform and a most likely faster AF induction. The pacing-time required to achieve self-sustained AF varied among horses, suggesting a variation in AF vulnerability. Atrial pacing led to increased AF vulnerability and increased AF duration in all horses; this is the main feature of atrial remodelling where “AF begets AF” (2). One horse (#6) required a particularly long pacing-time, which could be attributed to its small size (408 kg), as AF is difficult to induce in small horses (11). Despite the prolonged period before persistent AF was induced in this horse, intensive pacing did seem to remodel the heart, as this horse could not be pharmacologically cardioverted by flecainide on day 55.

### Electrical remodelling

A number of studies on electrical properties in horses with induced AF have been published (5, 7–9) and in one of them, spontaneous cardioversion to SR allowed refractory period measurements without drug interferences (8). The equine atrial refractoriness is rate-dependent and decreases with AF (7–9) in a similar way to humans (21), dogs (22), pigs (23) and goats (2). Shortening of the refractory period allows more fibrillatory waves to co-exist, which further promotes AF. In this study, we measured the rate of the fibrillatory waves while the horses were in AF. Several studies have validated AFR as a measurement of electrical remodelling (24–26), and this method allowed us to study the progression of electrical remodelling non-invasively and without drug interference. We found a significant increase in AFR between day 1 and day 55, which is representative of electrical remodelling. We have previously studied AFR in horses with both short-term induced AF and spontaneous persistent AF (26). Similar to the findings in this study, the AFR in horses with induced short-term AF was lower (269 ± 36 fpm) compared to horses with spontaneous persistent AF (364 ± 26 fpm) (26). These values correspond well with AFR values in patients with paroxysmal and persistent AF, respectively (25, 27).

Electrical remodelling also describe changes in the genes encoding ion channels during AF. In this study, no difference in ion channel expression was observed between the AF and control group. Other studies have found a down-regulation in the L-type Ca^2+^ channel, the α subunit of cardiac Na^+^ channels and Kv4.3 channel (28, 29), however, as these analyses are underpowered similar conclusions cannot be made from the present study.

### Functional remodelling

Atrial fibrillation is also known to cause atrial enlargement and contractile dysfunction that can persist several days after cardioversion (30). In patients, atrial contractile dysfunction, also known as “atrial stunning”, is considered an important risk factor for thromboembolisms (31). In this study, we observed a significant reduction in atrial function (LA-Fac_active_) three days after AF induction was initiated. Atrial remodelling has also been documented in dogs after 6 weeks of continuous pacing (22) and a study on instrumented goats tachypaced into AF, showed that atrial function was reduced already after 5 min of AF and that the reduction increased with AF duration (32). Similar results have been described for horses with spontaneous (18) and induced AF (8, 33).

### Ventricular function

Atrial fibrillation is associated with impaired ventricular function and heart failure is a common comorbidity in AF patients. Although not fully elucidated, this is likely to be the result of a tachycardia-induced cardiomyopathy caused by a persistently increased HR (34). In human patients, the increased HR is often managed with rate-control drugs such as beta-blockers (1). Other animal models also have increased HR, and these are often treated medically with digoxin, or surgically with an AV nodal ablation (20). Horses have a high vagal tone, and if they develop persistent AF they usually have a normal to slightly increased HR (35). In accordance with this, we found no significant difference in HR between the AF and control group. In addition, no changes in ventricular size or function from the echocardiographic examinations were found and no differences in ventricular fibrosis between control and AF group were observed. These results are in accordance with observations from the equine clinic, where horses with spontaneous AF rarely develop heart failure without underlying cardiac disease (18, 36). Tachycardia induced heart failure albeit medical rate control has been shown in pigs and dogs, while goats did not show signs of heart failure. Increased myocardial fibrosis was also evident in the ventricles of the pigs and dogs but not in the goats (20).

Cardioversion to sinus rhythm.

The success rate of medical cardioversion to SR decreases as AF progresses (1). A human clinical study previously reported that the cardioversion success rate decreased from 86 to 22% if the patients treated with flecainide had been in AF for longer than 10 days (37). We found similar results in this study, as the cardioversion success rate fell from 100% on days 3 and 9 to 33% on day 55. This suggests that the horse could be an interesting model for testing novel antiarrhythmic drugs, as it seems that the response to medical treatment is comparable.

### Structural remodelling

In addition to electrical and functional remodelling, AF is also known to cause structural remodelling, which includes hypertrophy of the cardiomyocytes, myolysis, alterations in connexin expression and interstitial fibrosis, all of which increase conduction heterogeneity and support the maintenance of AF (30, 38). To our knowledge, this is the first study to compare structural remodelling in horses with and without AF and we found significantly increased amounts of collagen in the AF group. The difference between groups was more pronounced in the left atrium compared to the right atrium, where only a tendency was present. Several studies in both human patients (39) and animal models (20) show an increase in collagen as a consequence of AF. The literature seems to agree on increased fibrosis in the left atrium in patients with AF, yet there are mixed results regarding right atrial fibrosis (40). Some studies report an equal amount of fibrosis in both the left and right atrium, while other studies report similar findings as this study, where less fibrosis was observed in the right atrium compared to the left (41–43). Atrial fibrosis increases with age which have been suggested to be a contributing factor to the increased AF burden in the aging population (44). In this study two out of the three control horses were above the age of 15 years which might have caused an increase in fibrosis in the control group.

### Limitations

This study is limited by the relatively low number of animals included. The small sample size is strengthened by the longitudinal design of the study which facilitates repeated measurements, however, both the AF and SR measurements holds several missing values. The missing AF values are attributed to the prolonged AF induction, which could have been avoided by continuous burst-pacing. Sinus rhythm measurements were limited by the attenuated ability of flecainide to cardiovert longstanding AF. Future studies should consider either a more potent drug than flecainide or electrical cardioversion in order to obtain SR measurements. AFR only provides a summary of the atrial activity and local heterogeneity cannot be captured with this method. Technical errors prevented us from assessing local changes in AFCL and atrial refractoriness. Future studies should explore endo- and epicardial atrial recordings and mapping of fibrillation patterns.

## Conclusion

In this study, we have described a model for chronic AF in horses which are naturally predisposed to AF. We observed electrical remodelling (as AFR increased during the study period), functional remodelling (described by atrial enlargement and reduced left atrial fractional area change), and structural remodelling (as the atria in the AF group were more fibrotic than in the control group), which are findings likely to contribute to the maintenance of AF. In accordance with this, a decrease in the efficacy of the anti-arrhythmic drug flecainide was observed. No functional remodelling of the ventricles or signs of heart failure were observed. This makes the model interesting for long-term AF studies.

## Supplementary information

**Additional file 1.** “Figure”: Word document with all figures in high quality. “Supplementary material”: Detailed description of how the echocardiographic ventricular variables were analysed, detailed description of tissue harvest, preparation of tissue post mortem and quantitative reverse transcription polymerase chain reaction (qPCR, Table S1). In addition, the results of ion channel expression (Fig. S1), and echocardiographic measurement (Table S2–4) are shown.

## Data Availability

The datasets used and/or analysed during the current study are available from the corresponding author on reasonable request.

## Abbreviations

2D: Two-dimensional echocardiography
AF: Atrial fibrillation
AFCL: Atrial fibrillation cycle length
AFR: Atrial fibrillatory rate
AMM: Anatomical M-mode
ECG: Electrocardiography
HR: Heart rate
LA: Left atria
LAA: Left atrial appendage
LAA_a_: Left atrial area at onset of the P wave
LAA_max_: Left atrial area at mitral valve opening
LAA_min_: Left atrial area at mitral valve closure
LAD: Left atrial diameter at mitral valve opening
LA-Fac_active_: Active left atrial fractional area change
LA-Fac_passive_: Passive left atrial fractional area change
LA-Fac_total_: Total left atrial fractional area change
LV: Left ventricle
QTc: Corrected QT interval
RA: Right atrium
RAA: Right atrial appendage
SR: Sinus rhythm

## Footnotes

1 AssurityTM MRI 2272, St. Jude Medical, St. Paul, MN

2 Tendril STS Pacing Leads 100 cm, St. Jude Medical, St. Paul, MN

3 TambocorTM, Meda A/S, Allerød, Denmark

4 Televet 100, Telemetric ECG, Kruuse A/S, Langeskov, Denmark

5 Cardiolund AFR Tracker software, cardiolund.com, Lund Sweden

6 Vivid I, 3S Phased Array transducer, GE Healthcare, Horten, Norway

7 EchoPAC PC, GE Healtcare, Horten, Norway

8 Axio Scan Z1 slide scanner, Zeiss, Oberkochen, Germany

9 ZEN 2.3 Blue edition, Zeiss, Oberkochen, Germany

10 ZEN Intellesis, 2017, Zeiss, Oberkochen, Germany

11 Microsoft, Redmont, 2016, USA

12 GraphPad Software, San Diego, CA, USA
