# Supplementary material for: Longitudinal study of electrical, functional and structural remodelling in an equine model of atrial fibrillation
Source: BMC Cardiovasc Disord. 2019 Oct 21;19:228. doi: 10.1186/s12872-019-1210-4 (PMC6805623; doi:10.1186/s12872-019-1210-4)
Supplement: Supplementary file 1 — Additional file 1. “Figure”: Word document with all figures in high quality. “Supplementary material”: Detailed description of how the echocardiographic ventricular variables were analysed, detailed description of tissue harvest, preparation of tissue post mortem and quantitative reverse transcription polymerase chain reaction (qPCR, Table S1). In addition, the results of ion channel expression (Fig. S1), and echocardiographic measurement (Table S2–4) are shown. [file 12872_2019_1210_MOESM1_ESM.docx]

**Supplementary material**

**Longitudinal Study of Electrical, Functional and Structural Remodelling in an Equine Model of Atrial Fibrillation**

Short title: Chronic Equine Model of AF

Eva Zander Hesselkilde^a^, DVM, PhD, Helena Carstensen^a^, DVM, PhD, Mette Flethøj^a^, DVM, PhD, Merle Fenner^a^, DVM, Ditte Dybvald Kruse^b^, MSc, Stefan M. Sattler^c,d^, MD, MSc, Jacob Tfelt-Hansen^c,e^, MD, PhD, Steen Pehrson^c^, MD, PhD, Thomas Hartig Braunstein^b^, MSc, PhD, Jonas Carlson^f^, MSc, PhD, Pyotr G. Platonov^f^, MD, PhD, Thomas Jespersen^b^, DSc, PhD, Rikke Buhl^a^, DVM, PhD

^a^ Department of Veterinary Clinical Sciences, Faculty of Health and Medical Sciences, University of Copenhagen, Højbakkegaard Allé 5, 2630 Taastrup, Denmark. ^b^ Department of Biomedical Sciences, Faculty of Health and Medical Sciences, University of Copenhagen, Blegdamsvej 3, 2200 Copenhagen, Denmark. ^c^ Department of Cardiology, The Heart Centre, Copenhagen University Hospital, Blegdamsvej 9, 2100 Copenhagen, Denmark. ^d^ Department of Medicine I, University Hospital Munich, Campus Grosshadern, Ludwig-Maximilians University Munich (LMU), Munich, Germany. ^e^ Department of Forensic Medicine, Faculty of Medical Sciences, University of Copenhagen, Denmark. ^f^ Arrhythmia Clinic, Skåne University Hospital and Department of Cardiology, Clinical Sciences, Lund University, 21185 Lund, Sweden.

Email addresses:

Eva Zander Hesselkilde: [evah@sund.ku.dk](mailto:evah@sund.ku.dk), Helena Carstensen: [hc@sund.ku.dk](mailto:hc@sund.ku.dk), Mette Flethøj:

[flethoj@sund.ku.dk](mailto:flethoj@sund.ku.dk), Merle Fenner: [merle.fenner@sund.ku.dk](mailto:merle.fenner@sund.ku.dk), Ditte Dybvald Kruse: [ditte.kruse@sund.ku.dk](mailto:ditte.kruse@sund.ku.dk), Stefan M. Sattler: [sattler@sund.ku.dk](mailto:sattler@sund.ku.dk), Jacob Tfelt-Hansen: [jacob.tfelt@regionh.dk](mailto:jacob.tfelt@regionh.dk), Steen Pehrson: [Steen.Pehrson@regionh.dk](mailto:Steen.Pehrson@regionh.dk), Thomas Hartig Braunstein^:^ [thobra@sund.ku.dk](mailto:thobra@sund.ku.dk), Jonas Carlson: [Jonas.carlson@med.lu.se](mailto:Jonas.carlson@med.lu.se), Pyotr G. Platonov: [pyotr.platonov@med.lu.se](mailto:pyotr.platonov@med.lu.se), Thomas Jespersen: [thojes@sund.ku.dk](mailto:thojes@sund.ku.dk), Rikke Buhl: [rib@sund.ku.dk](mailto:rib@sund.ku.dk)

Corresponding author: R. Buhl, Department of Veterinary Clinical Sciences, Faculty of Health and Medical Sciences, University of Copenhagen, Højbakkegaard Allé 5, 2630 Taastrup, Denmark, (+45) 29217782, rib@sund.ku.dk

**Material and Methods:**

*Echocardiography*

Ventricular variables measured to assess ventricular size and function, are listed below. From Anatomical M-mode (AMM): interventricular septum measured at the end of the diastole (onset of the R wave, IVSd), interventricular septum measured peak systole (IVSs), left ventricular internal diameter measured at the end of the diastole (LVIDd), left ventricular internal diameter measured at peak systole (LVIDs), left ventricular free wall measured at the end of the diastole (LVFWd) and left ventricular free wall measured at peak systole (LVFWs). Three additional variables were calculated: mean wall thickness at the end of the diastole (MWTd) = (IVSd+LVFwd)/2, relative wall thickness at the end of the diastole (RWTd) = (IVSd+LVFWd)/LVIDd and fractional shortening (FS) = (LVIDd-LVIDs)/LVIDd*100.

From the two-dimensional (2D) plane the following were calculated: left ventricular end-diastolic volume (LVEDV), left ventricular end-systolic volume (LVESV), ejection fraction (EF) = LVEDV-LVESV)/LVEDV, stroke volume (SV) = LVEDV-LVESV and cardiac output (CO) = SV*HR.

*Tissue harvest and preparation of the tissue*

After the study period the horses were euthanized by bolt-stunning and within minutes the heart was removed and perfused with a cooled cardioplegic solution ([1](#_ENREF_1)). With a 4 mm biopsy punch cardiac tissue for microscopy was harvested from the left atrial appendage (LAA), right atrial appendage (RAA) and right ventricle (RV). Additional tissue for ion channel expression was harvested from the left ventricular epicardium (LVepi), left ventricular mid-myocardium (LVmid) and left ventricular endocardium (LVendo).

Biopsies for microscopy were fixated in 4% paraformaldehyde for one week before transferred to 70% ethanol and stored at room temperature until further analysis. Before staining the specimens were dehydrated by an increasing series of ethanol (70-99.9%) followed by Tissue-Clear® Xylene substitution^[[1]](#endnote-1)^ and paraffin-embedding. Sections of 4 µm were cut on an HM 355S automatic rotary microtome^[[2]](#endnote-2)^ and mounted on glass slides. Sections were deparaffinized by a descending series of ethanol (99.9-70%), followed by Tissue-Clear® Xylene substitution and rehydrated in water. In accordance with the manufacturer’s protocol for connective tissue stain kit^[[3]](#endnote-3)^, glass slides were covered and incubated in Picro-Sirius Red solution for one hour, rinsed twice in 0.5 % acetic acid and rinsed three times in absolute ethanol before cover glass mounting with NeoMount®^[[4]](#endnote-4)^.

Tissue for ion channel expression studies was embedded in RNAlater^[[5]](#endnote-5)^, snap-frozen in liquid nitrogen and stored at -80°C until further analysis. Quantitative reverse transcription polymerase chain reaction (qPCR) was performed as previously described ([1](#_ENREF_1)) using TRI Reagent®^[[6]](#endnote-6)^ for purification, High-Capacity cDNA Reverse Transcription Kit^[[7]](#endnote-7)^ for cDNA synthesis and TaqMan Gene Expression for quantitative qPCR^[[8]](#endnote-8)^. We investigated the most prominent calcium, sodium and potassium ion channels responsible for the cardiac action potential (Table 1S, ([1](#_ENREF_1))). TaqMan® assays, specific for SCN5A, KCNH2, KCNJ3, KCNQ1, and KCNA5, were predesigned, whereas the equine sequences for KCNJ2, KCNJ5, KCNN1, KCNN2, KCNN3, KCND3, KCNIP3, and CACNA1C were manually designed using Primer Express® Software 3.0^[[9]](#endnote-9)^.

| Gene | Channel | Current |
| --- | --- | --- |
| SCNA5 | Nav1.5 | *I*_Na_ |
| CACNA1C | Cav1.2 | *I*_CaL_ |
| KCND3 | Kv4.3 | *I*_to_ |
| KCNIP2 | KChiP2 | *I*_to_ |
| KCNA5 | Kv1.5 | *I*_Kur_ |
| KCNH2 | Kv11.1 | *I*_Kr_ |
| KCNQ1 | Kv7.1 | *I*_Ks_ |
| KCNJ2 | Kir2.1 | *I*_K1_ |
| KCNJ3 | Kir3.1 | *I*_KACh_ |
| KCNJ 5 | Kir3.4 | *I*_KACh_ |
| KCNN1-3 | K_Ca_2.x | *I_KCa_* |

Table S1. Genes characterized with qPCR and their respective ion channels and currents

**Results**

*qPCR*

No significant differences were observed in ion channel expression between AF and control group for any of the 13 genes studied. Expression levels for each gene in the six locations studied are presented below (fig. S1).

Figure S1: Ion channel expression


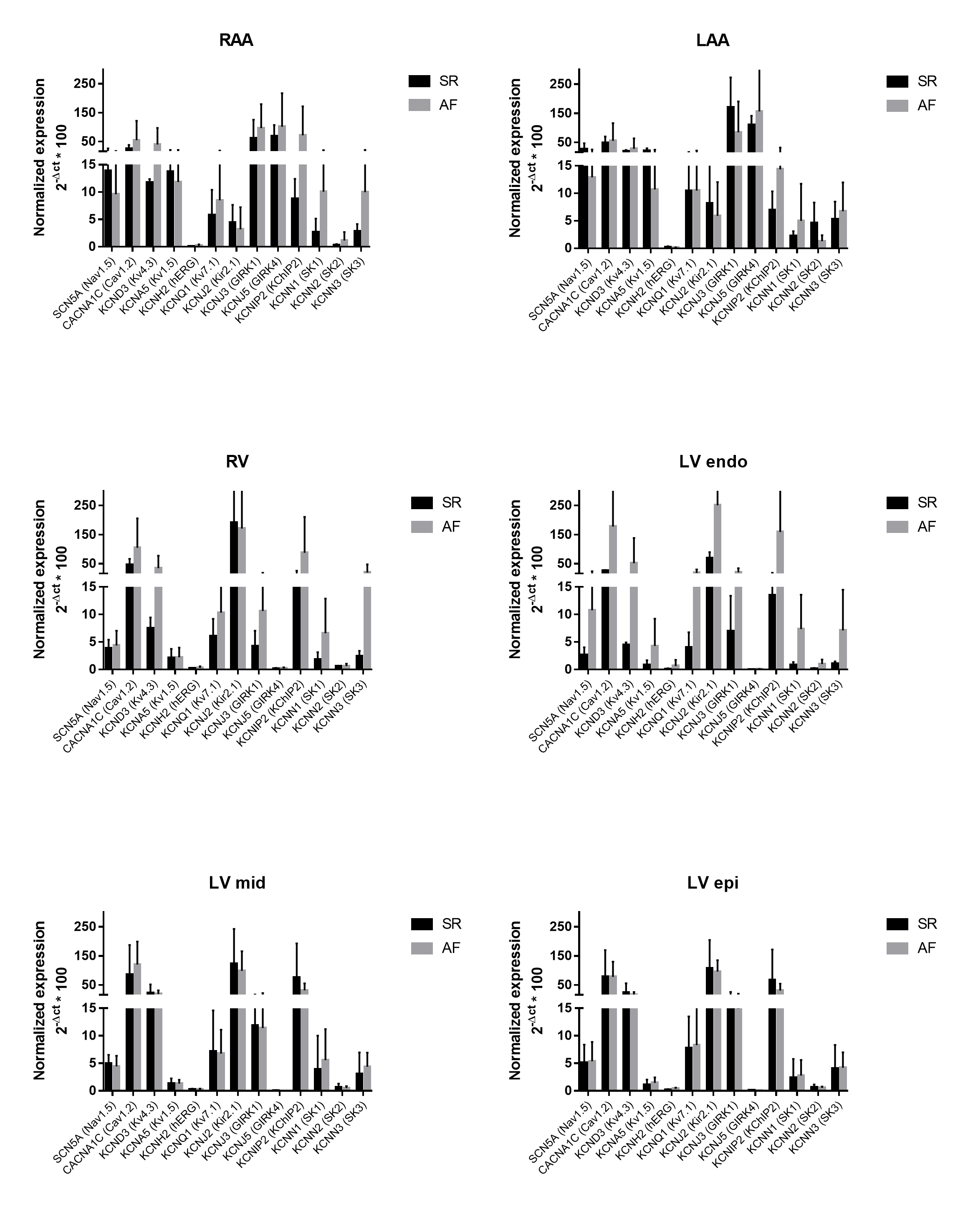


Figure S1: Quantitative RT-PCR results normalized to ACTB (β-actin) presented as 100 * 2^-ΔCt^ (mean±SD). AF: Atrial fibrillation, SR: sinus rhythm, RAA: right atrial appendage, LAA: left atrial appendage, RV: right ventricle, LV endo, LV mid, and LV epi: left ventricular endocardium, mid-myocardium and epicardium, respectively.

Table S2

| AF group (measured in SR) | | | | | | | |
| --- | --- | --- | --- | --- | --- | --- | --- |
| Left atrium | Unite | Before  n = 6 | Baseline  n = 6 | Day 3  n = 6 | Day 9  n =5 | Day 27  n = 5 | Day 55  n = 2 |
| LAA_min_ | cm^2^ | 53±4 | 54.5±5 | 55.0±5 | 64.8±6* | 63.3±4* | 66.6±2* |
| LAA_a_ | cm^2^ | 64.7±4 | 63.4±6 | 53.1±4* | 56.5±7 | 56.5±4 | 59±6 |
| LAA_max_ | cm^2^ | 83.5±10 | 81.3±3 | 73.8±7 | 84.3±4 | 75.9±6 | 83.2±7 |
| LAD | cm | 11.8±0.6 | 11.6±0.5 | 11.1±0.6 | 11.7±0.8 | 11.1±0.7 | 11.6±0.4 |
| LA-Frac_passive_ | % | 21.8±8 | 21.9±7 | 27.7±6 | 32.8±7 | 25.2±8 | 29.0±0.6 |
| LA-Frac_active_ | % | 18±7 | 13.8±7 | -3.6±5* | -15.0 ±11* | -12.4±8* | -13.1±7* |
| LA-Frac_total_ | % | 35.9±8 | 32.8±7 | 25.2±7 | 23.1±6 | 16.4±3* | 19.8±4 |
| **AF group** (measured during AF) | | | | | | | |
| Left atrium | Unite |  | Baseline  n = 5 | Day 3  n = 3 | Day 9  n =6 | Day 27  n = 6 | Day 55  n = 6 |
| LAA_min_ | cm^2^ |  | 53±4 | 54.8±3 | 53.1±4 | 60.5±7 | 63.4±4* |
| LAA_max_ | cm^2^ |  | 63.6±8 | 65.7±7 | 66.6±5 | 71.7±5 | 76.7±5* |
| LAD | cm |  | 11.1±1 | 10.8±0.8 | 10.8±0.8 | 11±1 | 11.2±0.8 |
| LA-Frac_total_ | % |  | 16.1±5 | 16.5±4 | 20.3±4 | 15.7±5 | 17.1±5 |
| **Control group** (measured in SR) | | | | | | | |
| Left atrium | Unite | Before  n = 3 | Baseline  n = 3 | Day 3  n = 2 | Day 9  n =3 | Day 27  n = 3 | Day 55  n = 3 |
| LAA_min_ | cm^2^ | 55.9±9 | 55.4±11 | 51.7±1 | 53.8±5 | 53±5 | 48.9±2 |
| LAA_a_ | cm^2^ | 59±5 | 59.1±4 | 58.4±1 | 60.3±1 | 58.8±5 | 53.9±2 |
| LAA_max_ | cm^2^ | 77.7±10 | 79.5±4 | 79.7±1 | 82.3±3 | 77.5±2 | 74.5±7 |
| LAD | cm | 11.6±0.8 | 11.7±0.9 | 11.4±0.1 | 12.3±0.2 | 11.3±0.4 | 11.1±1 |
| LA-Frac_passive_ | % | 23.7±6 | 25.8±2 | 26.7±3 | 26.6±3 | 24±8 | 27.1±9 |
| LA-Frac_active_ | % | 5.6±8 | 6.7±12 | 11.5±0.4 | 10.8±10 | 9.8±4 | 9.1±7 |
| LA-Frac_total_ | % | 28.2±2 | 30.8±10 | 35.1±2 | 34.7±5 | 31.5±8 | 33.9±8 |

Tabel S2: Measures of left atrial (LA) size and function by two-dimensional echocardiography (2DE), before the pacemaker implantation (before), at baseline (SR baseline = day 0, AF baseline = day 1) and at day 3, 9, 27 and 55 after atrial fibrillation (AF) was initiated. The following were measured: left atrial area at mitral valve closure (LAA_min_), left atrial area at onset of the P wave (LAA_a_), left atrial area at mitral valve opening (LAA_max_), left atrial diameter at mitral valve opening (LAD), passive left atrial fractional area change (LA-Frac_passive_), active left atrial fractional area change (LA-Frac_active_) and total left atrial fractional area change (LA-Frac_total_). SR: sinus rhythm. Data are presented as mean±SD. *Indicates significant difference from baseline.

Table S3

| AF group (measured during AF) | | | | | | | | |
| --- | --- | --- | --- | --- | --- | --- | --- | --- |
| Left ventricle | Unite | Baseline  n = 6 | Day 3  n = 2 | Day 9  n =6 | Day 27  n = 6 | Day 55  n = 6 |  |  |
| AMM |  |  |  |  |  |  |  |  |
| HR | min^-1^ | 48.7±6 | 49±8 | 52.1±6 | 51.6±4 | 47.5±5 |  |  |
| IVSd | cm | 3.6±0.4 | 3.6±0.4 | 3.7±0.4 | 3.4±0.3 | 3.3±0.2 |  |  |
| IVSs | cm | 4.4±0.6 | 4.3±0.7 | 4.5±0.5 | 4±0.4 | 4.1±0.4 |  |  |
| LVIDd | cm | 11.3±0.7 | 10.9±0.2 | 11.1±0.5 | 11.8±0.6 | 11.3±0.7 |  |  |
| LVIDs | cm | 7.7±0.5 | 7.4±0.3 | 8±0.6 | 8.4±0.5 | 7.9±1 |  |  |
| LVPWd | cm | 2.3±0.2 | 2.1±0.3 | 2.5±0.3 | 2.2±0.2 | 2.4±0.5 |  |  |
| LVPWs | cm | 3.3±0.4 | 3.3±0.5 | 3.3±0.4 | 3.1±0.1 | 3.4±0.5 |  |  |
| MWTd | cm | 3±0.3 | 2.8±0.4 | 3.1±0.3 | 2.8±0.3 | 2.8±0.2 |  |  |
| RWTd | cm | 0.5±0.1 | 0.5±0.1 | 0.5±0.1 | 0.5±0.1 | 0.5±0.1 |  |  |
| FS | % | 31.4±5 | 31.7±3.5 | 27.6±5.7 | 28.3±1.6 | 29.7±5.5 |  |  |
| LVD mass | g | 3834±609 | 3387±731 | 3952±634 | 3781±367 | 3594±400 |  |  |
| 2DE |  |  |  |  |  |  |  |  |
| HR | min^-1^ | 47.9±3 | 50±5 | 48.5±5 | 49±2 | 45.1±4 |  |  |
| LVEDV | ml | 880±165 | 896±33 | 886±97 | 956±215 | 1149±163 |  |  |
| LVESV | ml | 416±112 | 403±50 | 448±68 | 456±134 | 449±62 |  |  |
| EF | % | 53.3±5 | 54.9±7 | 49.6±4 | 52.2±9 | 56.9±6 |  |  |
| SV | ml | 463±63 | 493±83 | 438±46 | 500±133 | 600±132 |  |  |
| CO | l/min | 22.1±3 | 24.5±2 | 21.2±4 | 24.3±6 | 27.2±6 |  |  |

Table S3: Ventricular measurements for the AF group obtained in atrial fibrillation (AF). Measures of left ventricular (LV) size and function obtained by anatomical M-mode (AMM) and two-dimensional echocardiography (2DE), before the pacemaker implantation (before) and at baseline (day 0) and the following procedure days. Mean±SD. *Indicates significant difference from baseline. HR: heart rate, IVS: interventricular spetal thickness, LVID: left ventricular internal diameter, LVPW: left ventricular posterior wall. MWT: mean wall thickness, RWT: relative wall thickness, FS: fractional shortening, LVD mass: left ventricular mass, LVEV: left ventricular end volume, EF: ejection fraction, SV: stroke volume, CO: cardiac output, d: end-diastolic measure, s: peak systolic measure.

Table S4

| Control group (measured in SR) | | | | | | | |
| --- | --- | --- | --- | --- | --- | --- | --- |
| Left ventricle | Unite | Before  n = 3 | Baseline  n = 3 | Day 3  n = 2 | Day 9  n =3 | Day 27  n = 3 | Day 55  n = 3 |
| AMM |  |  |  |  |  |  |  |
| HR | min^-1^ | 36.7±8 | 37±3 | 36.5±8 | 38.9±1 | 39.6±5 | 43.5±12 |
| IVSd | cm | 3.4±0.1 | 3.5±0.5 | 3.9±0.6 | 3.4±0.7 | 3.5±0.5 | 3.7±0.3 |
| IVSs | cm | 4±0.4 | 4.5±0.4 | 4.8±0.7 | 4.6±0.1 | 4.4±0.5 | 4.6±0.4 |
| LVIDd | cm | 11.8±1 | 11.6±1.2 | 12.5±0.6 | 12.3±0.6 | 11.6±1 | 11.8±0.9 |
| LVIDs | cm | 8±1.2 | 7.5±1.6 | 8.3±0.5 | 7.8±0.9 | 7.7±0.6 | 7.7±0.5 |
| LVPWd | cm | 2.1±0.1 | 2.4±0.4 | 2.1±0.03 | 2.2±0.2 | 2.3±0.2 | 2.3±0.2 |
| LVPWs | cm | 3±0.7 | 3.3±0.7 | 2.8±0.3 | 3.2±0.4 | 3.3±0.2 | 3.1±02 |
| MWTd | cm | 2.8±0.1 | 2.9±0.1 | 3±0.3 | 2.8±0.4 | 2.9±0.3 | 3±0.3 |
| RWTd | cm | 0.5±0.03 | 0.5±0.05 | 0.5±0.03 | 0.5±0.05 | 0.5±0.03 | 0.5±0.05 |
| FS | % | 32.4±5 | 36.2±8 | 33.8±1 | 36.5±5 | 33.5±5 | 34.8±2 |
| LVD mass | g | 3669±598 | 3956±806 | 4562±1052 | 4105±950 | 3846±869 | 4186±802 |
| 2DE |  |  |  |  |  |  |  |
| HR | min^-1^ | 33.6±1 | 38.3±2 | 38.2±7 | 39.7±2 | 40.4±6 | 45±11 |
| LVEDV | ml | 1013±156 | 1074±201 | 1191±78 | 996±129 | 1094±181 | 1114±196 |
| LVESV | ml | 355±108 | 447±99 | 446±17 | 426±78 | 395±54 | 398±89 |
| EF | % | 65.5±6 | 58.5±1 | 62.5±1 | 57.4±2 | 63.6±4 | 64.5±2 |
| SV | ml | 658±49 | 575±68 | 745±61 | 569±52 | 699±139 | 716±110 |
| CO | l/min | 22.1 ±2 | 23.9±3 | 28.6±7 | 24.4±1 | 28±5 | 31.6±6 |

Table S4: Ventricular measurements for the control group, obtained in sinus rhythm (SR). Measures of left ventricular (LV) size and function obtained by anatomical M-mode (AMM) and two-dimensional echocardiography (2DE), before the pacemaker implantation (before) and at baseline (day 0) and the following procedure days. Mean±SD. *Indicates significant difference from baseline. HR: heart rate, IVS: interventricular spetal thickness, LVID: left ventricular internal diameter, LVPW: left ventricular posterior wall. MWT: mean wall thickness, RWT: relative wall thickness, FS: fractional shortening, LVD mass: left ventricular mass, LVEV: left ventricular end volume, EF: ejection fraction, SV: stroke volume, CO: cardiac output, d: end-diastolic measure, s: peak systolic measure.

**References**

1. Haugaard MM, Hesselkilde EZ, Pehrson S, Carstensen H, Flethoj M, Praestegaard KF, et al. Pharmacologic inhibition of small-conductance calcium-activated potassium (SK) channels by NS8593 reveals atrial antiarrhythmic potential in horses. Heart rhythm : the official journal of the Heart Rhythm Society. 2015;12(4):825-35.

1. Tissue- Clear® Xylene Substitute, Sakura Finetek Europe [↑](#endnote-ref-1)
2. HM 355S Automatic Rotary Microtome Thermo Fisher Scientific Inc. [↑](#endnote-ref-2)
3. Picro-Sirius Red, Abcam, Cambridge, UK [↑](#endnote-ref-3)
4. NeoMount®, Sigma-Aldrich, MO, USA [↑](#endnote-ref-4)
5. RNAlater, Sigma-Aldrich, MO, USA [↑](#endnote-ref-5)
6. TRI Reagent®, Sigma-Aldrich, MO, USA [↑](#endnote-ref-6)
7. Thermo Fisher Scientific, MA, USA [↑](#endnote-ref-7)
8. Thermo Fisher Scientific, MA, USA [↑](#endnote-ref-8)
9. Applied Biosystems®, Thermo Fisher Scientific, MA, USA [↑](#endnote-ref-9)
